# Supplementary material for: DNA methylation in cocaine use disorder–An epigenome-wide approach in the human prefrontal cortex
Source: Front Psychiatry. 2023 Feb 14;14:1075250. doi: 10.3389/fpsyt.2023.1075250 (PMC9970996; doi:10.3389/fpsyt.2023.1075250)
Supplement: Supplementary file 1 [file Data_Sheet_1.DOCX]

Supplementary Material


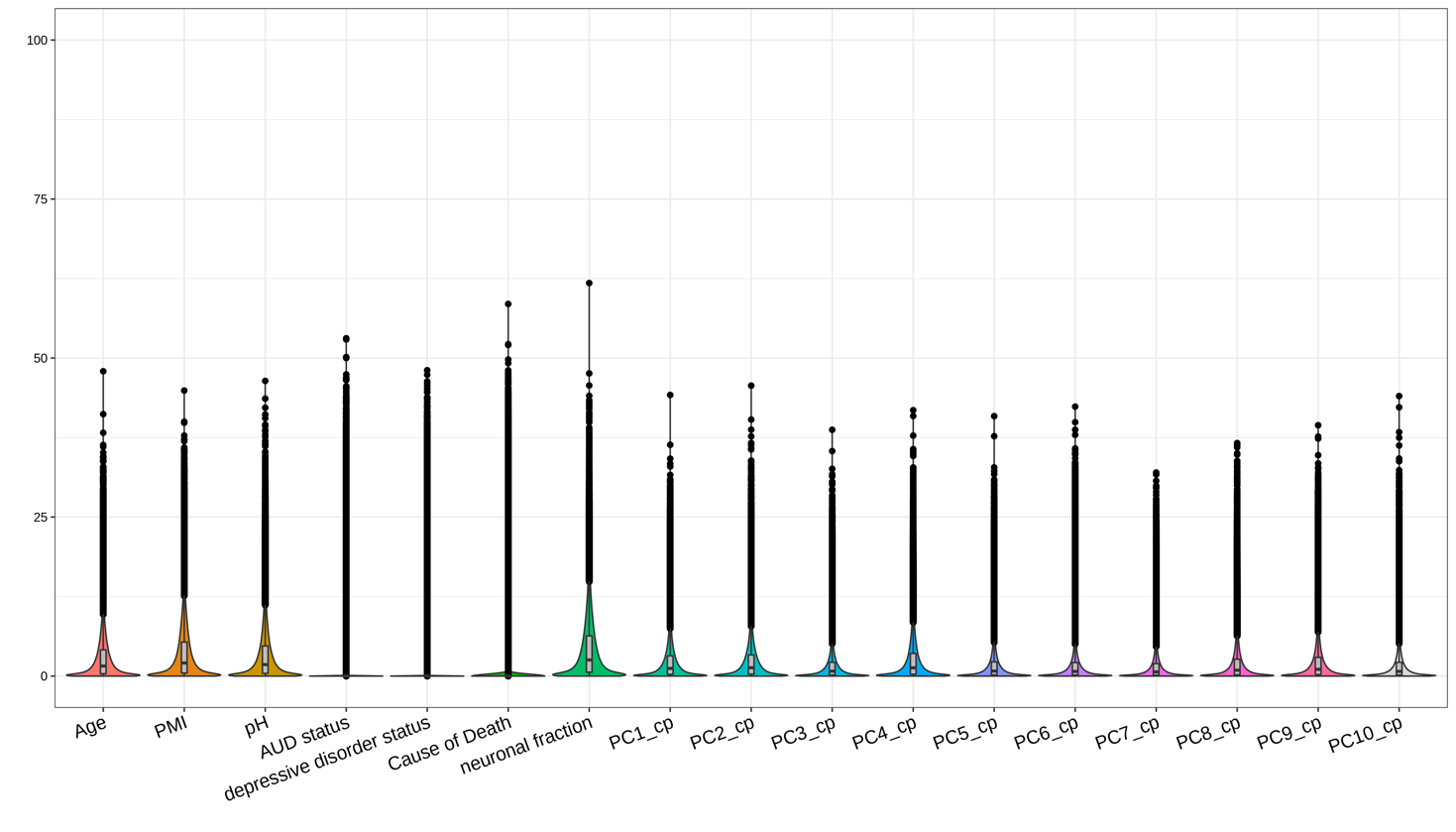


variance explained (%)

Supplementary Figure 1: results of the variance partition analysis confirming the selected covariates for the linear model in the epigenome-wide association study.


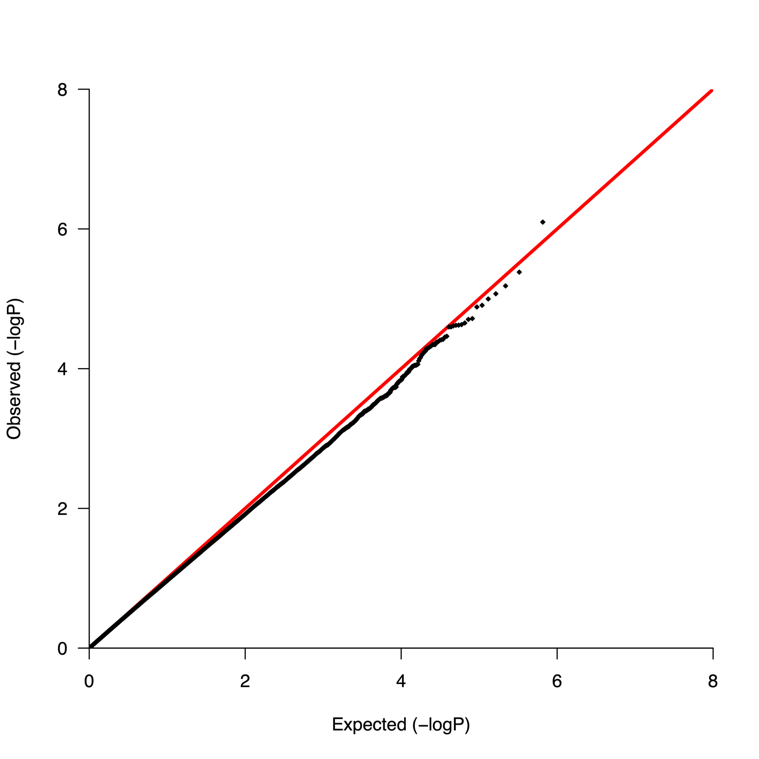


Supplementary Figure 2: QQ-Plot (λ = 0.969) of the EWAS of cocaine use disorder in human postmortem brain tissue of Brodmann Area 9 (N = 42).


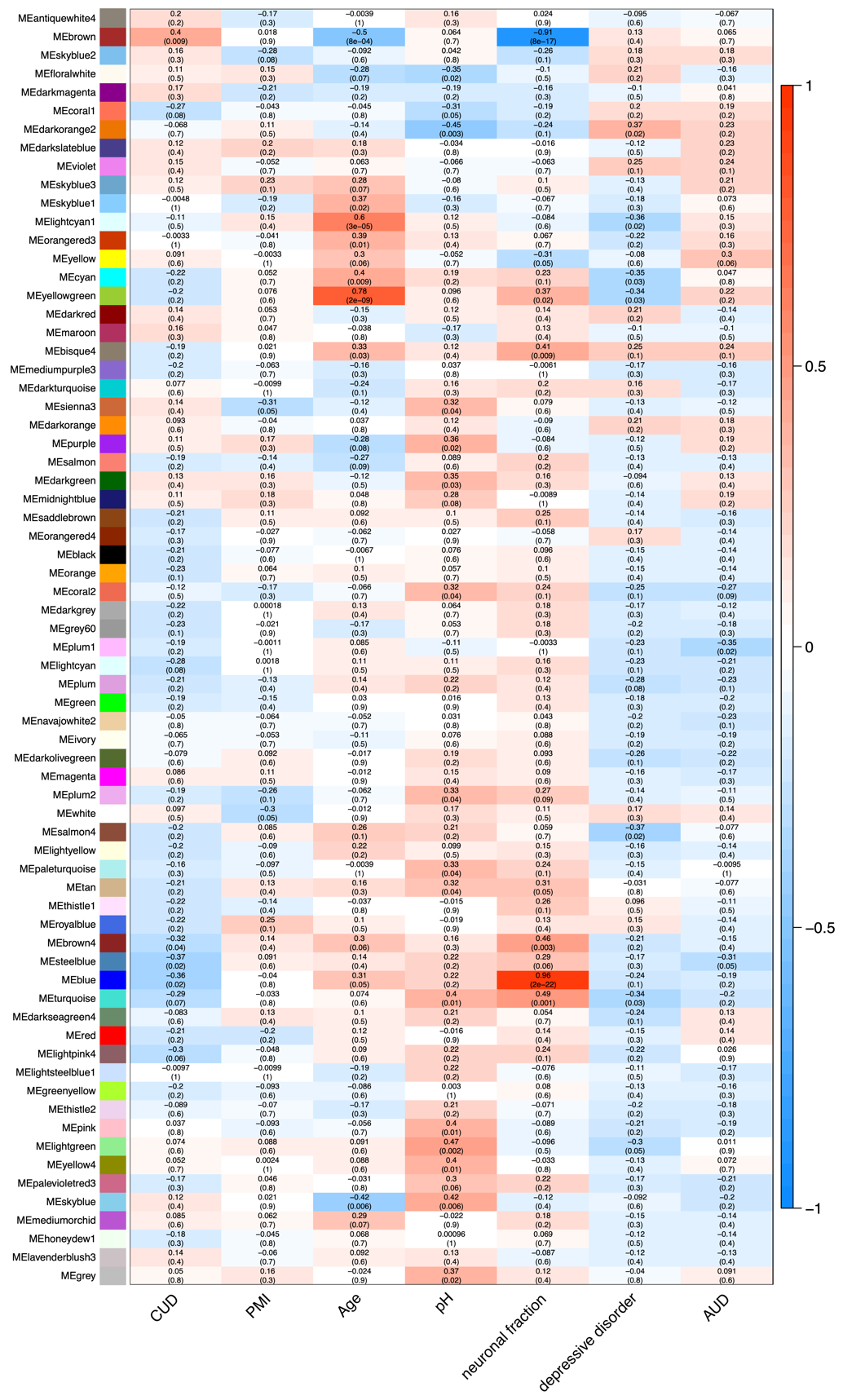


Supplementary Figure 3: module-trait correlation plot displaying all co-methylation modules resulting from WGCNA.

CUD: cocaine use disorder, PMI: postmortem interval, neuronal fraction: estimated neuronal cell type proportion, AUD: alcohol use disorder.


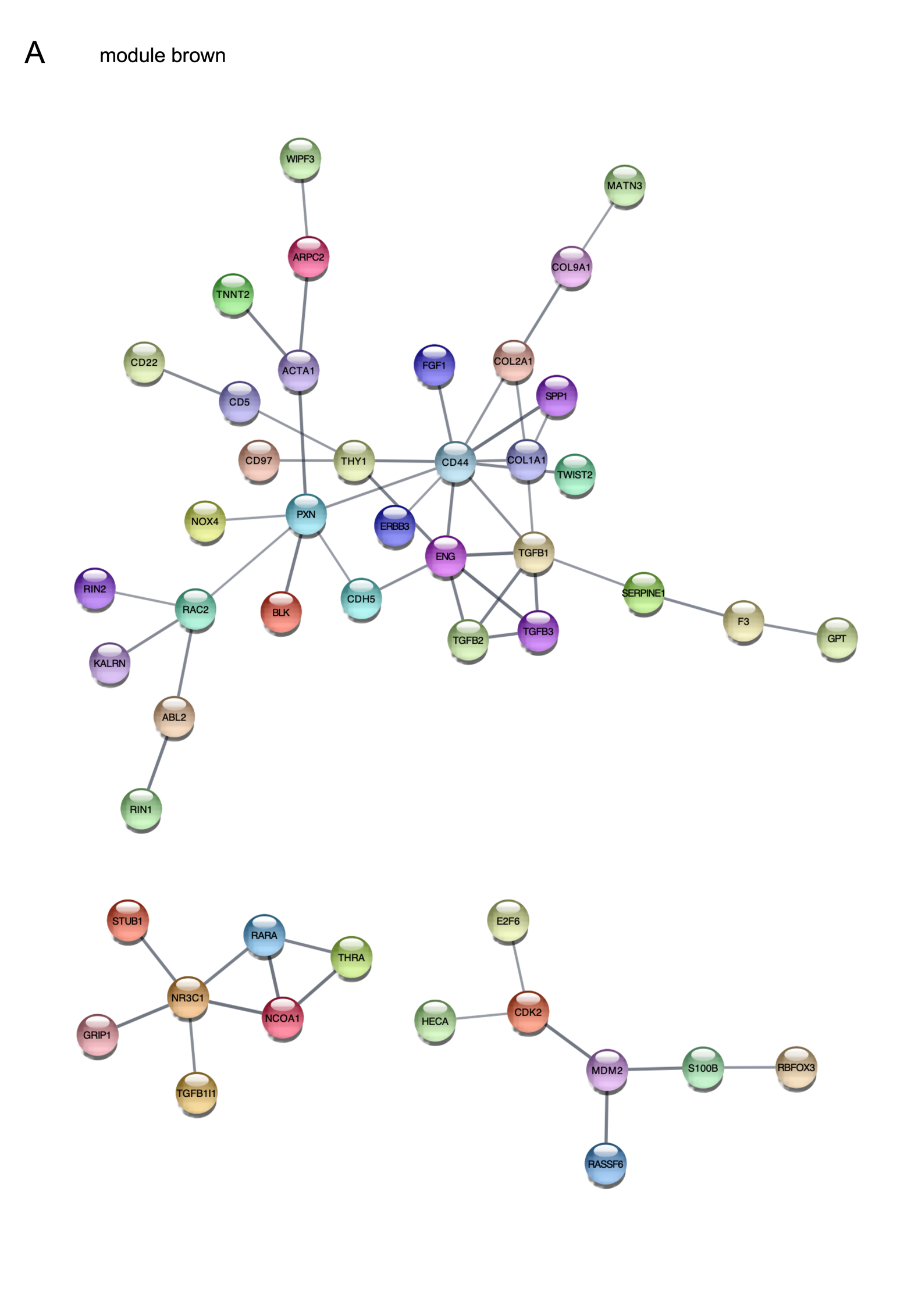


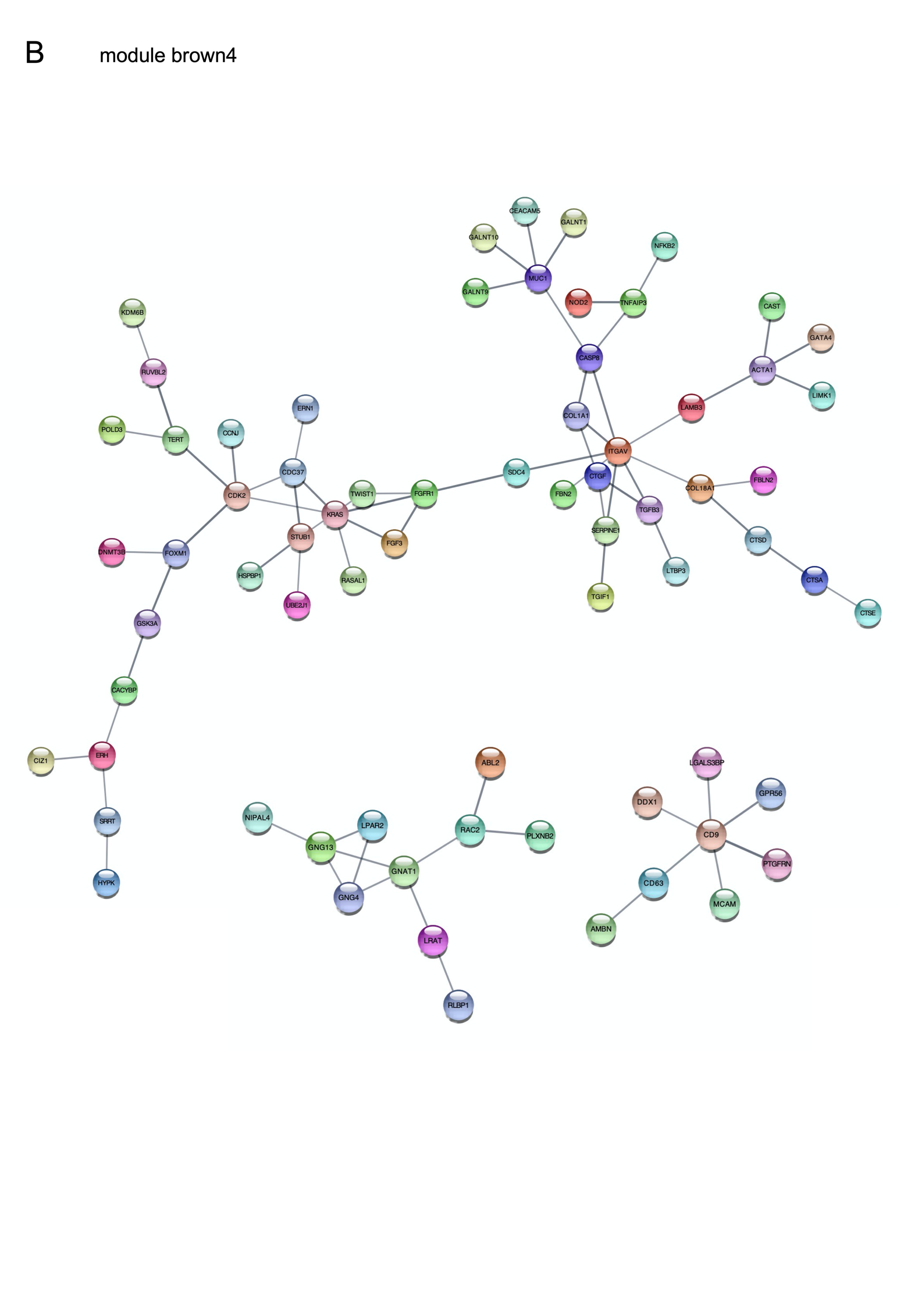


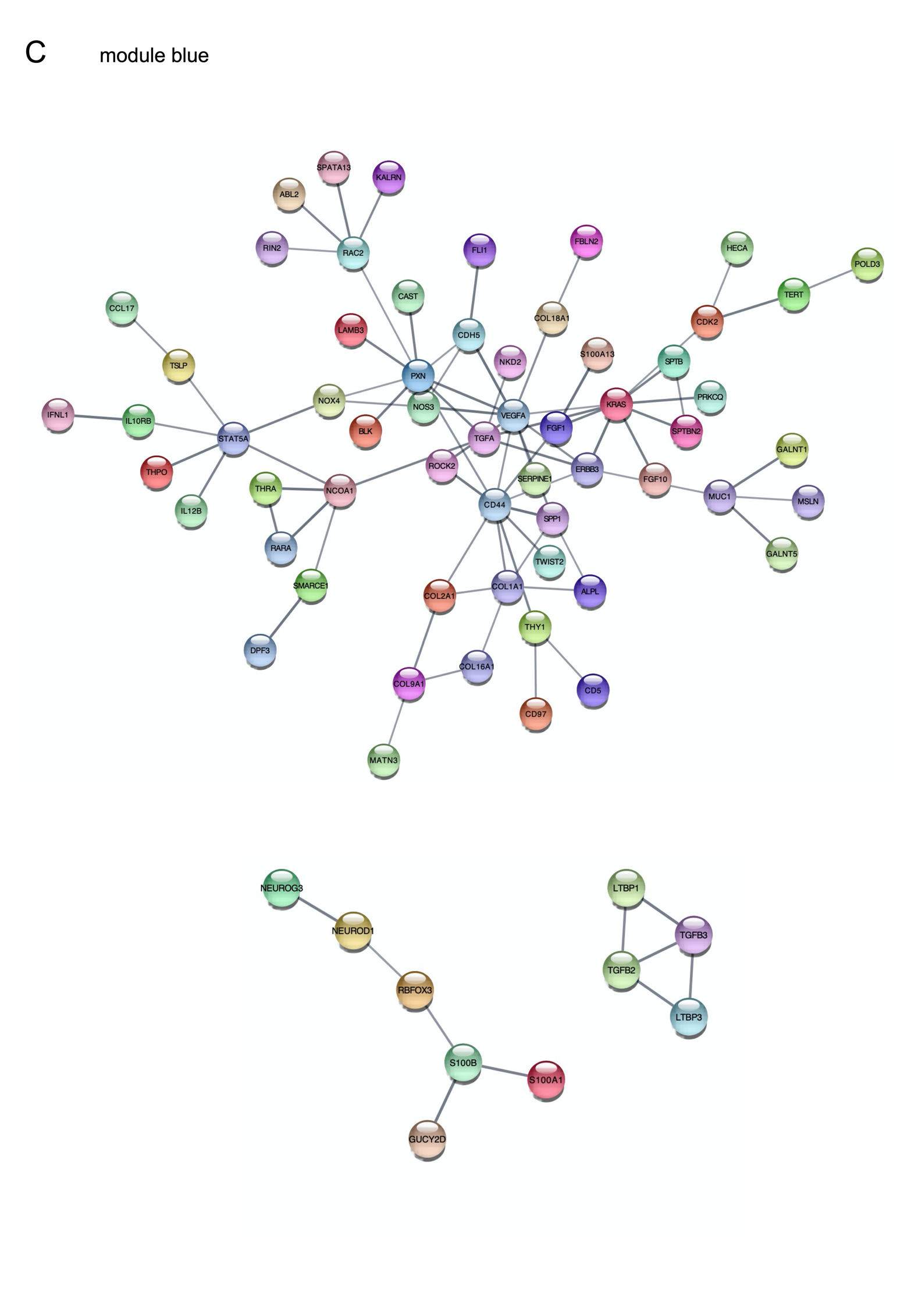


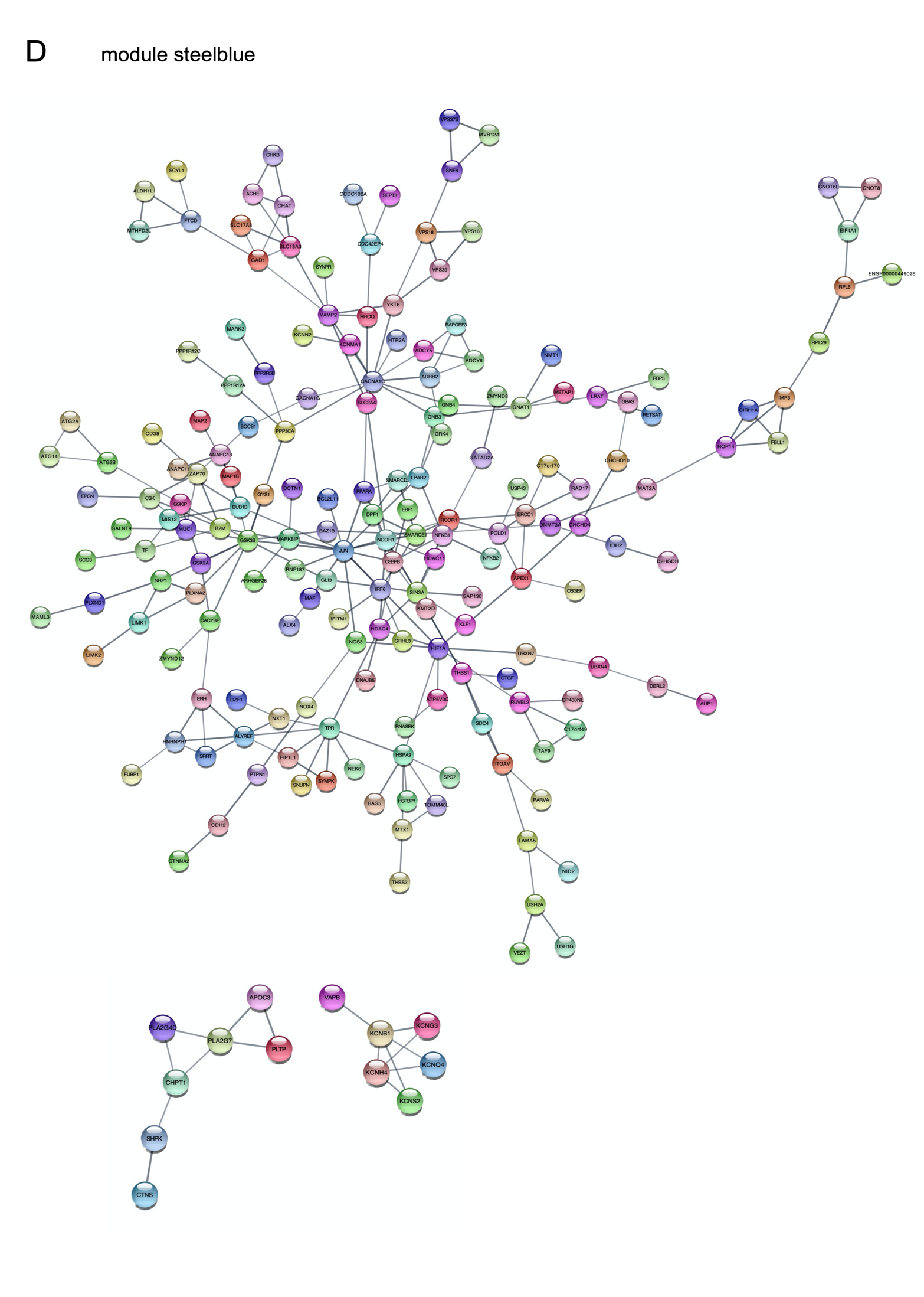


Supplementary Figure 4: protein-protein-interaction networks derived from CUD-associated co-methylation module hub genes.

The top three networks ranked by size and the connectivity of nodes are shown for WGCNA-derived co-methylation modules (A) brown, (B) brown4, (C) blue, and (D) steelblue. Network plots were generated using the *Search Tool for the Retrieval of Interacting Genes/Proteins* (STRING, v.11.5) with an interaction score threshold of 0.7 (high confidence interactions).
